# Association of extreme heat events with sleep and cardiovascular health: A scoping review

**DOI:** 10.21203/rs.3.rs-3678410/v1

**Published:** 2023-12-20

**Authors:** Nathan Ashe, Sarah Wozniak, Malcom Conner, Rayan Ahmed, Michelle R Demetres, Nour Makarem, Parissa Tehranifar, Rajalakshmi Nandakumar, Arnab Ghosh

**Affiliations:** Weill Cornell Medical College: Weill Cornell Medicine; Weill Cornell Medical College: Weill Cornell Medicine; Weill Cornell Medical College: Weill Cornell Medicine; Weill Cornell Medicine; S J Woods Library: Weill Cornell Medicine; Columbia University Irving Medical Center; Columbia University Irving Medical Center; Cornell Tech; Weill Cornell Medicine

**Keywords:** Extreme heat events, heat-related sleep disruption, cardiovascular health, health equity

## Abstract

**BACKGROUND:**

Extreme heat events (EHEs), driven by anthropogenic climate change, exacerbate the risk of cardiovascular disease (CVD), although the underlying mechanisms are unclear. Disturbances in sleep health, caused by excessive heat, may be one way EHEs increase the risk of incident or recurrent CVD. Our objective was to systematically review the empirical peer-reviewed literature on the relationship between EHEs, sleep health, and cardiovascular measures and outcomes, and narratively describe methodologies, evidence, and gaps in this area.

**METHODS:**

A comprehensive literature search was performed in the following databases from inception – June 2023: Ovid MEDLINE, Ovid EMBASE, CINAHL, Web of Science and The Cochrane Library. Studies retrieved were then screened for eligibility against predefined inclusion/exclusion criteria.

**RESULTS:**

Of the 2035 records screened, three studies met the inclusion criteria. Cardiovascular (CV) measures described included blood pressure (BP), heart rate (HR), and HR variability (no CVD outcomes were described) and objective and subjective measurements of sleep health outcomes included sleep duration, calmness, ease of falling asleep, ease of awakening, freshness after awakening, and sleep satisfaction. Two studies were controlled trials, and one was a cohort study. During EHEs, individuals slept for shorter periods of time and less efficiently, with greater degrees of HR variability in two of the three studies lasting at most 1–2 days; BP (both systolic and diastolic) significantly decreased during EHEs in two of the studies. No formal assessment of a mediating relationship between EHE exposure, sleep outcomes, and the CV measures was undertaken.

**CONCLUSIONS:**

There is a paucity of data that examines the link between CVD, sleep, and extreme heat as a possible mechanism of elevated CVD risk during EHEs, despite a strong physiological rationale. Further research is needed to empirically test this relationship rigorously as EHEs become more frequent and their deleterious impacts of health increase.

## Background

Extreme heat events (EHEs) are periods of unusually high temperatures, which are increasing in frequency, intensity, and duration as a result of anthropogenic climate change.^1^ While no single definition of EHEs exists,^2^ since 1950, the number of heatwave days (defined as at least three consecutive days above the 90th percentile of daily maximum temperature) are estimated to have increased by 2.26 per decade globally, while the cumulative heat (i.e., the extra heat produced by a heatwave over a given season) increased by 2.84 °C per decade.^3^ Heat exposure is associated with adverse cardiovascular (CV) events, with every 1°C rise in ambient temperature significantly raising the risk of cardiovascular disease (CVD)-related morbidity and mortality.^4^ Though increased CVD risk due to EHEs have been thoroughly documented in the literature, the mechanisms leading to associated CVD morbidity outcomes remain unclear.^5^

**Figure 1:**
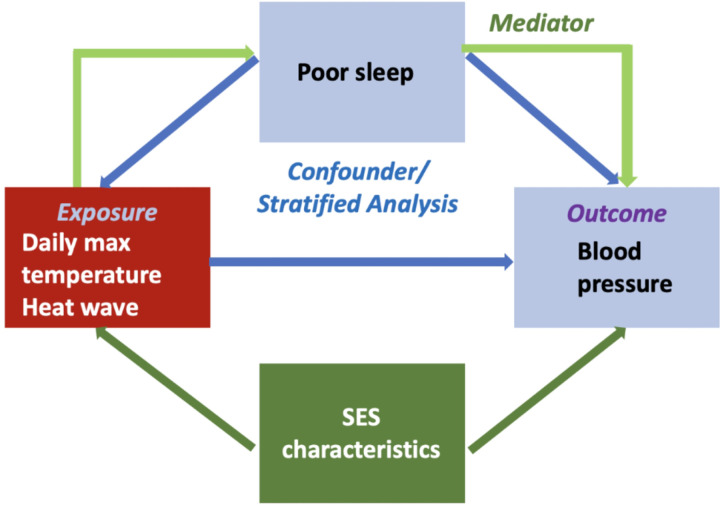
Hypothesized model of relationship between EHEs, sleep quality, and CV health.

Existing research describe a surge in cardiac output and hyperventilation during EHEs, yet the specific pathways leading to CVD exacerbation during EHEs are not fully delineated.^6,7,8^ One potential mechanism linking EHEs and CVD is sleep disruption. Rising temperatures have been associated with shorter sleep duration and poorer sleep quality, as has the aftermath of weather phenomena impacted by climate change like hurricanes, floods, and wildfires.^9,10,11,12^ Multiple dimensions of sleep health, including insufficient sleep duration, irregular sleep schedules, and poor sleep quality, can increase cardiometabolic risk predisposing to CVD.^13^ For instance, poor sleep has been linked to higher risk for hypertension,^14,15^ obesity,^16^ and type 2 diabetes^17^ via theorized mechanisms including inflammation^18,19^, glycemic dysregulation,^20^ increased sympathetic tone via increases in nocturnal catecholamines.^21^ Thus, this connection suggests a possible mechanism explaining the adverse impact of EHEs on CVD, with sleep as a mediating factor, as illustrated in Fig. 1.

Therefore, in this scoping review, we systematically examined the peer-reviewed literature that examined the relationship of EHEs with sleep health, and CV measures and CVD outcomes.

## Methods

This study was performed following the Preferred Reporting Items for Systematic Reviews and Meta-Analyses-Scoping reviews (PRISMA-ScR).^22^ In adherence to this statement, a protocol was registered with PROSPERO International prospective registry (PROSPERO; CRD42023432124).

### Search Strategy

A medical librarian (MRD) performed comprehensive searches to identify studies that examined the effect of EHEs (heat waves) on sleep health and CVD. Searches were completed on June 14, 2023 in the following databases: Ovid MEDLINE (ALL − 1946 to Present); Ovid EMBASE (1974 to present); CINAHL (EBSCO); Web of Science (Core Collection – Clarivate); and The Cochrane Library (Wiley). The search strategy included all appropriate controlled vocabulary and keywords for the concepts of “heat,” “sleep,” and “cardiovascular.” The full search strategies for all databases are available in an additional file. In order to limit publication bias in our initial search strategy, there were no language, publication date, or article type restrictions on the search strategy.

### Study Selection

**Figure 2:**
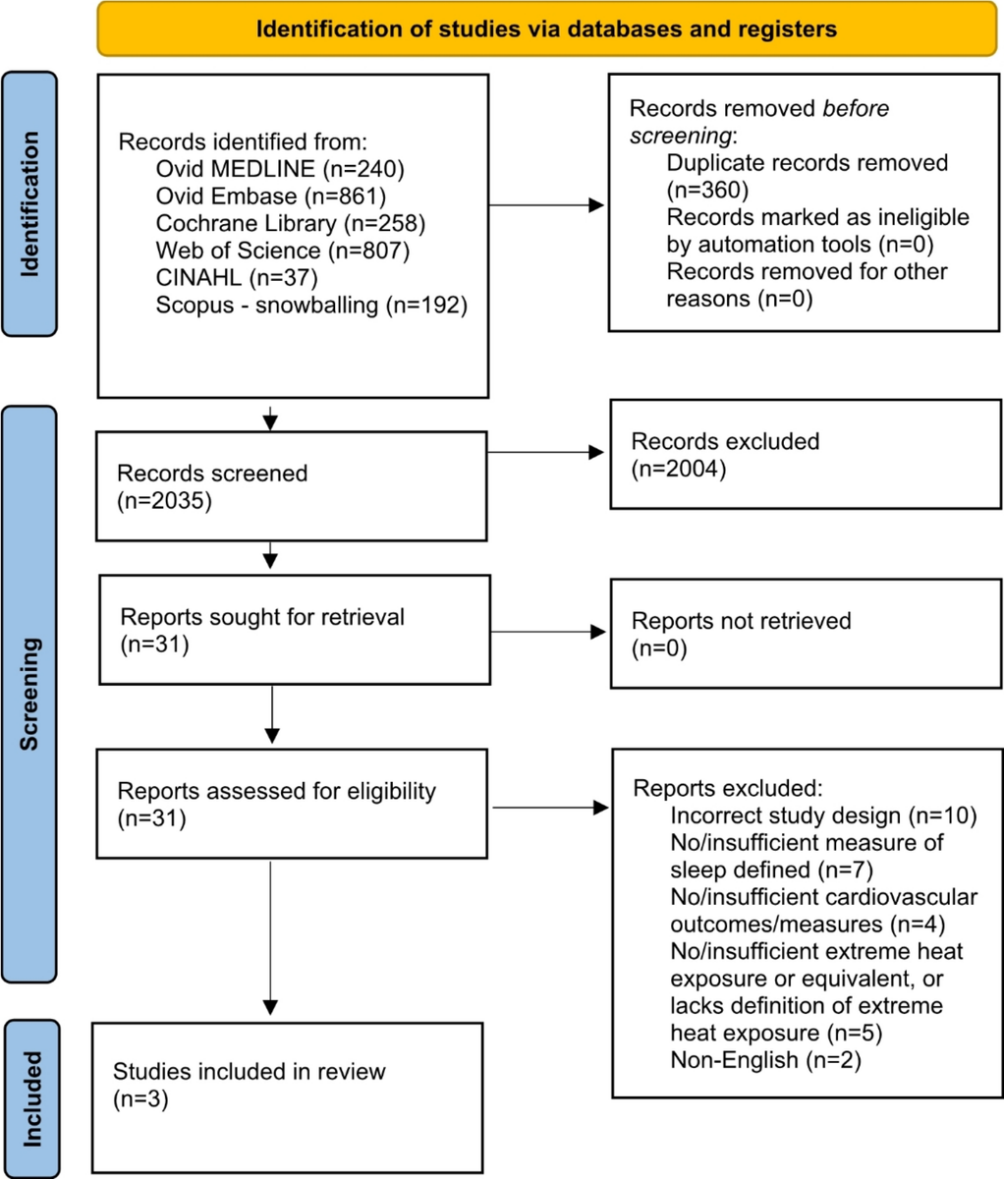
PRISMA-ScR Flow Diagram *From*: Page MJ, McKenzie JE, Bossuyt PM, Boutron I, Hoffmann TC, Mulrow CD, et al. The PRISMA 2020 statement: an updated guideline for reporting systematic reviews. BMJ 2021;372:n71. doi: 10.1136/bmj.n71

Retrieved studies were screened for inclusion using Covidence systematic review software. Titles and abstracts were reviewed against predefined inclusion/exclusion criteria by two independent reviewers. Discrepancies were resolved by consensus (NA, SW, RA, MRD, MC, AKG). For final inclusion, full text was then retrieved and also screened by two independent reviewers. Our inclusion criteria were articles that included the following: (1) EHEs, as defined by manuscript-specific definitions; (2) Reported sleep measures: sleep health disruptions (e.g., sleep duration ≤ 7 hours, irregular sleep, difficulty falling asleep, symptoms of sleep disorders, and/or daytime sleepiness); (3) CV measures (e.g., blood pressure, heart rate) and CVD events/diagnoses (e.g., diagnosis of hypertension, coronary artery disease, and peripheral arterial disease or acute CVD events such as myocardial infarction, stroke, and heart failure exacerbations); and (4) Adult participants (> = 18 years). Excluded studies were: (1) Non-English; (2) Review articles, commentaries, viewpoints, editorials, or case reports; (3) Insufficient CV measures or outcomes; (4) Insufficient measure of sleep defined; or (5) Lack of EHE or equivalent, or lacks definition of EHE. For articles selected for inclusion in this study, reference lists and citing articles were pulled from Scopus (Elsevier) and also screened. The full PRISMA flow diagram outlining the study selection process is presented as Fig. 2.

For more information, visit: http://www.prisma-statement.org/

### Data Extraction

Data extraction was performed by team members (NA, SW, MC, RA, AKG, MRD) independently in duplicate with predefined, standardized templates. Data points defined for extraction were: year; study location; study design; population under study; description of EHE/exposure; sleep outcome(s) evaluated; CV measure(s)/outcome(s) evaluated; and results.

## Results

### Summary of Articles

Three studies, summarized in Table 1, met criteria for inclusion in the analysis.

#### 1. Study Design

Two studies were controlled trials,^23,24^ while one was an observational cohort study.^25^ Huang et al. recruited participants (n = 41) into one of three intervention groups or a control group to assess the effect of these interventions (subsidies for air conditioning, education about health and environmental heat, and use of sprinklers to cool home exteriors) on sleep quality and CV measurements during a heatwave.^23^ Yan et al. performed a controlled, cross-over trial, placing participants (n = 16) in one of four room permutations, with rooms either 27°C or 30°C and either employing mechanical ventilation systems to circulate filtered outdoor air or not.^24^ Kim et al. observed the effects of a heat wave on elderly residents of rural communities in South Korea (n = 104).^25^

#### 2. Geographies covered and study settings

The three studies analyzed here report data from China,^23,24^ and South Korea^25^. One of these studies was at the regional level,^25^ while two were at the city level.^23,24^

#### 3. Extreme Heat Event Definitions

Definitions of EHEs varied greatly. In the Xinyi study, EHEs were defined as heatwaves using the 90th percentile daily maximum temperature.^23^ In the South Korea study, the criteria for heatwave were more than two consecutive days with a maximum temperature more than 33° C.^25^ Finally, the Shanghai controlled trial used indoor temperatures of 27° C and 30° C to model extreme heat exposure.^24^

#### 4. Sleep Outcomes Evaluated

Sleep outcomes used in the studies were both self-reported and objective. Subjective outcomes reported were self-assessed sleep duration,^25^ self-assessments of calmness, ease of falling asleep, ease of awakening, freshness after awakening, and sleep satisfaction.^24^ Objective outcomes were total sleep duration (split into deep sleep duration and light sleep duration) as measured by a smart band,^23^ and total sleep time, sleep efficacy (ratio of time asleep to time in bed), sleep onset latency (the time between turning off lights and falling asleep), time awake and duration of sleep stages (NREM sleep of stage N1, N2, and N3, and REM sleep), measured using electroencephalogram (EEG), bilateral electrooculogram (EOG), and chin electromyogram (EMG).^24^

#### 5. Cardiovascular Measures and Outcomes Evaluated

No CVD outcomes were evaluated in these studies. However, CV measurements were assessed. All CV measurements were assessed with objective data. These data included systolic (SBP) and diastolic blood pressure (DBP)^23,24,25^ measured with a sphygmomanometer and HR^23^ and HR variability^24^ measured with an electronic wrist monitor or ECG, respectively.

### Qualitative Synthesis:

None of the studies included in this review directly evaluated measures of sleep health as a mediator or confounder in the relationship between extreme heat and CVD, or CV measures. However, indirectly, Yan et al. showed a significant mean difference (MD) in HR variability (MD = 0.7 beats per minute [bpm]; p = 0.02) between 27°C and 30°C room at the same time as a significant decrease in total sleep time (MD = 39.1 minutes, p = 0.01), sleep efficiency (MD = 8%, p = 0.01), and REM sleep time (MD = 3.3 minutes, p = 0.05) and increase in time awake (MD = 38.1 minutes, p = 0.01) in rooms lacking mechanical ventilation (i.e., fans to bring in filtered outdoor air); as well as a decrease in sleep efficiency of 0.2% per increased bpm (p = 0.04) and an increase in time awake of 2.39 minutes per increased bpm (p = 0.04).^24^

Kim et al. present findings suggestive of a relationship between extreme heat and both sleep and CV measures, however these neither reached statistical significance nor implied that sleep played a mediating role between extreme heat and CV measures. DBP decreased significantly (p < 0.001) in subjects with hypertension, with a 1°C increase in indoor temperature decreasing DBP by 0.44 mmHg (95% CI: 0.04–0.84 mmHg). The association between indoor temperature and SBP was positive but not significant. The number of hours of sleep decreased with indoor temperature by 0.036 hours (95% CI: −0.138, 0.67 hours), however this result did not reach statistical significance.^25^

Similarly, Huang et al. show an association between extreme heat and both sleep and CV measures, though no causal mechanisms can be inferred. In the control group, DBP and SBP elevated from baseline during the heatwave, with SBP increasing significantly on days 1 and 2 by 5.33 mmHg (95% CI: 3.38–7.30; P = 0.01) and by 4.92 mmHg (95%CI: 2.74–7.09; P = 0.02), respectively. HR elevated from baseline and on day 1 and lowered to near-baseline by day 5. Deep sleep duration decreased significantly in the first 3 days by −0.48 h (95%CI: −0.61, −0.34; P = 0.00), −0.36 h (95%CI: −0.51, −0.21; P = 0.01), and − 0.25 h (95%CI: −0.37, −0.12; P = 0.05), respectively.^23^

## Discussion

This scoping review identified three articles examining the relationship between extreme heat, sleep, and CV health. These papers did not elucidate a mechanism linking extreme heat to worsening CV measures or CVD. Their findings are, however, largely in concordance with previous literature indicating that extreme heat adversely affects CV health, notably elevating BP,^4,5^ and that it lowers measures of sleep quality.^11,12^

Importantly, our review highlighted several current research challenges linking climate-amplified EHEs, sleep health, and CVD health. First, the studies that met our inclusion criteria suggest potential associations between heat, sleep, and CVD, yet fall short of delving into the underlying physiological mechanisms that might explain these associations. Second, the duration of the included studies was limited, the longest being 19 days.^25^ The limited follow-up period after the defined EHEs may constitute an underlying bias of these studies, given research suggests adverse CVD outcomes can last up to 2 weeks after the antecedent EHE event.^26,27^ Further research involving a longer follow-up post EHE could help elucidate the connection between sleep and CV health, examining the time course of potential returns to baseline. Indeed, such studies could provide insight into opportunities to examine acclimatization strategies. Third, while duration and quality of sleep were examined, future research could evaluate multiple dimensions of sleep health and their relationship to EHEs and CVD, including timing and alertness. Last, studies lacked the inclusion of participants from at-risk populations, such as those with underlying CVD or whose surrounding environments render them particularly exposed to extreme heat. Research including these populations could offer perspective on ways to aid those most vulnerable to harm in a warming climate.

Furthermore, the existing studies were limited in its geographic reach and lacked standardization of key terminology such as EHE definition. Given that all of the studies reviewed were conducted in East Asia, our analysis underscores the need for a broader geographic representation in research on this subject. Further analyses may show important regional variation: given that the impacts and manifestations of climate change are so variable, associations between EHEs, sleep, and CVD should be investigated in different settings to inform interventions and policy; and, research conducted in specific countries may find an association between EHEs, sleep, and CVD more likely, particularly where CVD outcomes are more prevalent.^28^ The need for a uniform characterization of what constitutes an EHE or heatwave is also important in order to promote coherence and comparability between studies.^2^ This would facilitate a more nuanced understanding of the broader implications of EHEs on health on a global scale, and allow comparisons across regions and countries.

Interestingly, the role of air conditioning (AC) as a potential confounder in the relationship between EHEs, sleep health, and CVD is likely an area for further study. In the studies conducted by Huang^23^ and Yan,^24^ a significant emphasis is placed on the role of interior room temperatures, positing them as more critical determinants of health outcomes than external temperatures. Their findings hint at the potential benefits of regulating indoor temperatures to enhance both sleep quality *and* CV health, thereby mitigating some of the adverse effects of EHEs. Notably, Huang’s research delineates that daytime exposure to heat does not suffice to induce elevated SBP, suggesting that nocturnal temperature levels particularly may play a pivotal role – an area of future research.^29^

The role of AC further highlights the important relationship between EHEs and health inequities. Sleep health disparities have been shown to explain up to 50% of cardiometabolic disparities in some studies.^30^ EHEs may exacerbate health inequities, especially given that populations in low resource settings may not have infrastructure to adapt to extreme heat. One example of this is in New York City, where there exists a 10 percentage point gap in the rates of AC scarcity between White and Black residents.^31^ Future research on the relationship between EHEs, cardiovascular health, and sleep health could examine the role of health inequity and accordingly suggest policy interventions to reduce inequities in both sleep and CVD outcomes, particularly in relation to EHEs.

Although we conducted a comprehensive review, the final number of results in very small. We argue that this underscores the early stage of research in this area, highlighting the need for more extensive and in-depth studies to build a robust body of evidence that can inform policy and public health interventions effectively to improve sleep, and cardiovascular health in the age of climate change.

We describe two limitations to our work. First, we did not include the non-English literature and therefore may have missed important peer-reviewed manuscripts. This is particularly noteworthy because of the three studies that met inclusion criteria, all were from countries outside the English-speaking regions. Second, we did not consider grey literature. However, given the nuanced nature of our research question and the conceptual framework we examined (Fig. 1), the peer-reviewed literature is the most likely source of empirical studies on this topic.

## Conclusion

In conclusion, this scoping review examined the peer-reviewed literature that described the relationship between EHEs, sleep health, and CV health, an area of research that is still in its infancy. The existing literature, though limited, underscores the pivotal role of interior temperatures in influencing health outcomes, hinting at the potential of targeted interventions to mitigate adverse effects. However, the current body of research is characterized by geographical limitations, and a lack of uniform definitions and conceptual framework to understand the connections between sleep, heat, and CV measurements, all of which necessitate a more globally representative and cohesive approach in future studies. Moreover, the exploration of the underlying physiological mechanisms and the development of effective interventions remain largely untapped areas. Understanding these dynamics could suggest public health policies and strategies that mitigate the increasingly frequent effects of climate change in general, but particularly on CV health.

## Figures and Tables

**Table 1:** Summary of Studies

First Author, Year	Geographic region	Study design	Population	Description of Extreme Heat Event	Sleep outcomes	CV measures/outcomes	Results	Comments
Huang, 2022	Xinyi, Xuzhou, China	Randomized Controlled Trials	n = 41; 60% female, Mean age = 58.8 years	Participants were split into three groups of 10 and one of 11, one control and three experimental. Baseline health metrics were taken on a normal-temperature day. For five days during a subsequent heatwave, three groups each received one of the following interventions: education about health during heatwaves; subsidies for electricity costs of running an AC or fan; or daytime water spraying of homes in an attempt to cool interior temperatures	Use of an unspecified smart band that measured TSD, DSD, and LSD. The paper did not specify how the smart band measured these durations	A wrist blood pressure monitor measured DBP and SBP and HR of participants. Both metrics were measured three times every morning for the five-day study period	In the control group, DBP and SBP elevated from baseline during the heatwave, with SBP increasing significantly on days 1 and 2 by 5.33 mmHg, (95% CI: 3.38–7.30; P = 0.01) and by 4.92 mmHg (95%CI: 2.74–7.09; P = 0.02), respectively. HR elevated from baseline and on day 1 and lowered to near-baseline by day 5. DSD decreased significantly in the first 3 days by −0.48 h (95%CI: −0.61, −0.34; P = 0.00), −0.36 h (95%CI: −0.51, −0.21; P = 0.01), and - 0.25 h (95%CI: −0.37, −0.12; P = 0.05), respectively. In the cooling-spray group, SBP increased significantly on day one by 3.18 mmHg (95%CI: 1.73, 4.63; P = 0.03) and on day two by 3.34 (95%CI: 1.76, 4.93; P = 0.04) before gradually declining and returning to baseline. DSD was reduced significantly on day 2 by −0.21 h (95%CI: −0.31, 0.11; P = 0.05).	The experimental group interventions were not fully described. Information on occupation and AC use was not collected in an initial questionnaire, nor was information on ability to pay for AC, which would shed light on the efficacy of subsidies.
Kim, 2022	Rural areas in southern South Korea: Gijang, Busan; Imsil, Jeollabuk-do; Gwangyang, Jeollanam-do; and Namhae, Gyeongsangnamdo	Cohort study	n = 104; 72.1% female, Mean age = 79.6 years	All participants were exposed to the 2018 heat wave in South Korea (August 119). Indoor temperature and relative humidity were measured twice a day (morning and afternoon) for three days. Outdoor temperature and relative humidity were retrieved from the Korean Meteorological Administration website for each study area, with average values between 9 AM through 12 PM used as morning data and 1 PM through 5 PM used as afternoon data	Number of hours of sleep during the prior night was self-reported to investigators on days in which temperature, relative humidity, and health measures were taken	Body temperature measured by infrared thermometer; DBP and SBP measured twice (morning and afternoon) per day using a sphygmomanometer	DBP decreased significantly (p < 0.001) in subjects with hypertension, with a 1 °C increase in indoor temperature decreasing DBP by 0.44 mmHg (95% CI: 0.04–0.84 mmHg). The association between indoor temperature and SBP was positive but not significant. Number of hours of sleep decreased with indoor temperature by 0.036 hours (95% CI: −0.138, 0.067 hours), however this result did not reach statistical significance.	No analysis was performed to determine if subjects who reported fewer hours slept had significant differences in BT, DPB, or SBP.
Yan, 2022	Shanghai China, in controlled hospital bedroom setting	Controlled, cross-over trial.	n = 16, 50% female, mean age 72 years	Each participant was assigned to one of four experimental conditions established in a 2×2 experimental design: hospital-based bedrooms were heated to either 27° or 30° C, and used or did not use a mechanical ventilation system to provide the room with filtered outdoor air. Participants spent 5 non-consecutive nights (1 adaptive and 4 observed) sleeping in their assigned room, with a 3-day interval between experimental nights	Objective: Total sleep time, sleep efficacy, sleep onset latency, time awake and duration of sleep stage, measured using EEG, bilateral EOG, and chin EMG Subjective: questionnaire each morning measuring calmness of sleep, ease of falling asleep, ease of awakening, freshness after awakening and sleep satisfaction on 5-point scales	Heart rate and heart rate variability measured by ECG; DBP and SBP measured before and after sleep using a sphygmomanometer	Compared to 27°C, individuals at 30°C had a significantly increased time awake (MD = 15.9 min in MV setting, MD = 38.1 min in NMV setting, p = 0.01), less total sleep time (MD = 14.5 min in MV setting, MD = 38.1 min in NMV setting, P = 0.01), less sleep efficiency (MD = 3% in MV, 8% in NMV, P = 0.01), and less REM sleep (MD = 7.4 min in MV setting, MD = 3.3 min in NMV setting, P = 0.05); heart rate variable significantly differed (MD = 0.7 bpm in MV setting; 0.7 bpm in NMV setting, P −0.02), DBP (P = 0.01) and SBP (P < 0.001) significantly decreased with increases in duration of deep sleep defined by REM	Rigorously designed experimental study

*Abbreviations:* AC, air conditioning; TSD, total sleep duration; LSD, light sleep duration; LSD, light sleep duration; REM, rapid eye movement; DBP, diastolic blood pressure; HR, heart rate; SBP, systolic blood pressure; EEG, electroencephalogram; EOG, electrooculogram; EMG, electromyogram; MV, mechanical ventilation; NMV, non-mechanical ventilation; MD, mean difference

## Data Availability

All data generated or analyzed during this study are included in this published article and its supplementary information files.

## Abbreviations

AC: Air Conditioning
BP: Blood Pressure
CV: Cardiovascular
CVD: Cardiovascular Disease
DBP: Diastolic Blood Pressure
EEG: Electroencephalogram
EHE: Extreme Heat Event
EMG: Chin Electromyogram
EOG: Bilateral Electrooculogram
HR: Heart Rate
MD: Mean Difference
MRD: Medical Librarian
PRISMA-ScR: Preferred Reporting Items for Systematic Reviews and Meta-Analyses-Scoping reviews
SBP: Systolic Blood Pressure
